# Development of a clinical pathway for behavioural and psychological symptoms of dementia care: A tool to improve resident outcomes

**DOI:** 10.1111/ajag.13093

**Published:** 2022-05-31

**Authors:** Kate J. Kennedy, Marion Eckert, Deborah Forsythe, Joanne Wagner, Greg Sharplin

**Affiliations:** ^1^ Rosemary Bryant AO Research Centre University of South Australia Adelaide South Australia Australia; ^2^ Eldercare Inc. Payneham South Australia Australia

**Keywords:** aged, critical Pathways, dementia, evidence‐based practice, patient‐centred care

## Abstract

**Objectives:**

Clinical pathways are used to improve the quality of care, reduce variation and maximise health or treatment outcomes in selected populations. The aim of this study was to develop a draft clinical pathway based on the best practice evidence for use in the management of behavioural and psychological symptoms of dementia (BPSD) in residential aged care facilities (RACFs).

**Methods:**

The pathway was developed using the best practice evidence from clinical practice guidelines, operational guides and a systematic literature review. A multidisciplinary team of health professionals and researchers worked in an iterative process to contextualise the proposed pathway to local needs and context, and improve its clarity and user‐friendliness. The pathway was then re‐assessed for accuracy and adherence to the evidence.

**Results:**

The draft pathway outlines processes for BPSD prevention, watchful waiting for mild‐to‐moderate BPSD, and specific interventions for severe BPSD. Ongoing risk assessment is required throughout, and non‐pharmacological options are first‐line interventions. Person‐centred care was found to be an important care component across all three phases. An instruction guide with colour‐coded flow charts was developed to assist staff with determining the best care and treatment for each person living with dementia. Feasibility testing is underway.

**Conclusions:**

A draft clinical pathway based on clinical practice guidelines was developed to enhance the translation of evidence into practice for the management of BPSD, by nursing and clinical leaders in RACFs.

**Policy Impact**

This paper describes the development of a draft clinical pathway based on the current best practice evidence to prevent or manage the onset of behavioural and psychological symptoms of dementia (BPSD) in residential aged care facilities (RACFs). This pathway first needs to be tested for feasibility, then pilot‐tested for usability and generalisability across multiple sites.

## 1 INTRODUCTION

In Australia, dementia is the second leading cause of death with one in 10 people over 65 years, and three in 10 people older than 85 years diagnosed with this disease.^1^ Approximately 90% of people with dementia will experience behavioural and psychological symptoms of dementia (BPSD), and the prevalence of mild‐to‐severe BPSD is estimated to be 61% of people living with dementia.^2^ A behavioural or psychological symptom of dementia is any behaviour exhibited by a person, which causes stress, worry, risk of or actual harm (physical or emotional) to themselves or others. Symptoms can include aggression, apathy, anxiety, agitation, psychotic symptoms, depression, disinhibited behaviours, wandering, nocturnal disruption and vocally disruptive behaviours.^3^ The BPSD can occur at different levels of severity with the stages of disease progression; the seven‐tiered model of BPSD developed by Brodaty and colleagues^2^, ^3^, ^4^ demonstrates the wide range of symptoms and severity encompassed by the term BPSD.^3^, ^5^ Residential aged care facilities (RACFs) are key providers of support to those experiencing more severe forms of BPSD,^2^, ^4^ which means they must ensure robust, evidence‐based practices for managing BPSD. Clinical practice guidelines provide this evidence in a highly comprehensive form; however, that comprehensiveness makes these documents large and can make implementability and accessibility challenging in clinical settings.^6^

Clinical pathways are based on clinical practice guidelines to improve quality of care, reduce variation and maximise health and treatment outcomes in targeted populations.^7^, ^8^ At present, there are no evidence‐based clinical pathways for the management of BPSD specific to nurses and other care workers in Australian RACFs.^9^ Furthermore, care deficiency issues have been highlighted in some RACFs across Australia recently (see the Oakden inquiry^10^ and the Royal Commission into Aged Care Quality and Safety,^11^, ^12^ regarding the gaps in operationalising safe and effective care for people living with dementia in Australia). Finally, the Australian Aged Care Standards (introduced in 2019) placed the consumer at the centre of their care and reinforced the need for using evidence‐based practice when providing care to consumers.^13^ The research team worked with a local aged care provider to develop a clinical pathway that would ensure that best practice, person‐centred care was delivered consistently in their RACFs. Full details of the rationale behind this project, the evidence that went into informing this pathway and the current state of dementia care in Australian RACFs have been discussed elsewhere.^9^

**FIGURE 1:**
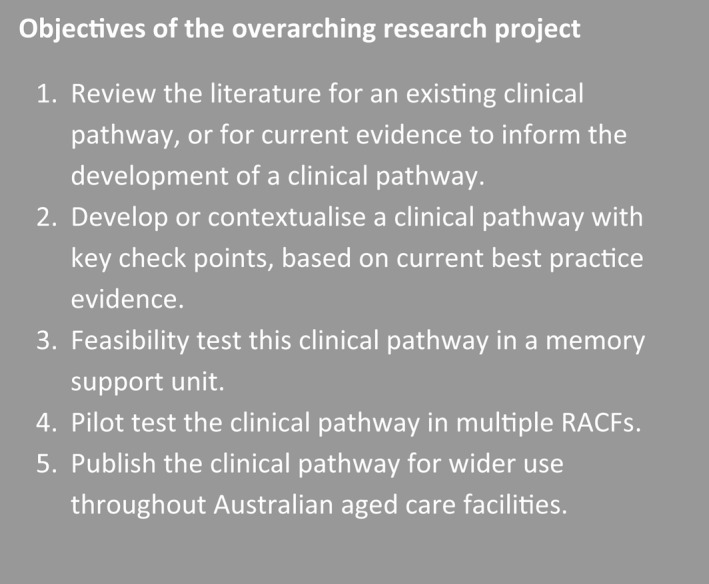
Objectives of the overarching research project^9^

This study is part of a larger research project (Figure 1) that aimed to develop a clinical pathway based on the current best evidence, providing key checkpoints for staff caring for those living with dementia and experiencing BPSD in RACFs. This paper outlines the development of a draft clinical pathway that will be tested for feasibility in RACFs.

In phase 1 of this study,^9^ a rapid review following PRISMA guidelines^14^ was conducted to find clinical practice guidelines, systematic reviews, or tools, protocols, pathways or guides that provided evidence‐based instruction for the prevention or management of BPSD in RACFs. The review identified 509 unique publications, seven of which fitted the inclusion criteria; three were specific to the Australian setting, but none provided a clinical pathway for the operationalisation of the current best practice evidence.^9^ To fill this gap, this paper focuses on phase 2 of the overarching research project (Figure 1) and describes the development process of a draft clinical pathway with key checkpoints for use in the management of BPSD by nurses in RACFs. This initial pathway was developed in conjunction with a multidisciplinary team from Eldercare Inc. and will be trialled for feasibility and implementability before pilot testing in multiple RACFs.

## 2 METHODS

### 2.1 Contributing publications

The literature identified in phase 1 of this study^9^ was used to develop a clinical pathway for use in the management of BPSD by nurses and care workers in RACFs. The core of the information included in the clinical pathway was based on recommendations from the *Clinical Practice Guidelines and Principles of Care for People with Dementia*,^3^ an Australian clinical practice guideline. This clinical practice guideline was adapted to Australian health‐care settings in 2016, from a UK clinical practice guideline on dementia care. The UK clinical practice guideline was further updated in 2018,^15^ and this newer version was used to check the currency of the Australian clinical practice guideline recommendations. This content was supplemented by national and international health service operational guides,^6^, ^16^ a clinical practice guideline developed for Dementia Behaviour Management Advisory Service (DBMAS) providers,^2^ a systematic review that provided updated data on the effectiveness of multisensory stimulation^17^ and a Canadian clinical practice guideline on deprescribing antipsychotics for BPSD.^18^

### 2.2 Ethics

This project 202613 has been approved by the Human Research Ethics Committee of the University of South Australia.

### 2.3 Multidisciplinary development team

A multidisciplinary team was formed to develop and conceptualise the evidence to the local setting. This team included researchers (with backgrounds in nursing, public health, occupational therapy and psychology) and health‐care professionals (site manager, clinical care consultant, clinical care manager, clinical leaders, the on‐call GP and the on‐call pharmacist). The allied health team and the chaplain at the site also provided feedback.

### 2.4 Development of the clinical pathway

The elements identified in the literature as critical to the evidence‐based management of BPSD provided by nurses and care staff in RACFs were collated into process stages based on risk management and care principles for BPSD (Table 1). These stages were as follows: risk assessment; prevention (no or low risk); watchful waiting (mild‐to‐moderate risk); escalation (high risk); and pharmacological interventions.

**TABLE 1:** Summary of the evidence that informed the development of the pathway stages

Phase of the pathway (colour code)	A brief summary of evidence
Risk assessment *(Green)*	A comprehensive health assessment, including a thorough medical review, should be conducted on admission, on change in the level of risk or at the initiation of changes in the person's behaviour, to detect any general health problems that may impact on quality of life, well‐being or other symptoms.^2^, ^3^, ^6^, ^15^, ^16^
Prevention (no or low risk) *(Green)*	The preferred approach is to minimise the development or impact of symptoms by providing person‐centred care. Person‐centred care is based on understanding the person's history and experiences (e.g. work, hobbies, family, environment, cultural and religious beliefs), their likes and dislikes, and taking their perspective into account. It is also important to ensure that the person has the opportunity for human contact and warm relationships with others.^2^, ^3^, ^6^, ^15^, ^16^, ^17^ Care plans should be developed in collaboration with the person with dementia and their family or nominated decision‐maker, should support the person's ability to be involved in decisions about their care, and should provide education and psychosocial support to the person with dementia and their family.^2^, ^6^, ^15^, ^16^
Watchful waiting (mild‐to‐moderate risk) *(Amber)*	Where risks or behaviour severity is mild or moderate (i.e. not causing serious distress or harm), a comprehensive assessment should be carried out as standard in response to any change in behaviour, and non‐pharmacological approaches to care should be the first line of intervention. Changes in treatment and care and the use of behavioural management interventions could avoid the use of antipsychotic drugs in people with dementia.^2^, ^3^, ^6^, ^15^, ^16^
Escalation (high risk) *(Red)*	Immediate action is required if the risk or behaviour severity is high (occurring frequently, causing serious distress or harm to self or others). Interventions to prevent harm can include referral to acute or specialist services, and/or the swift introduction of non‐pharmacological and pharmacological approaches to risk mitigation.^3^, ^6^, ^15^, ^16^, ^18^ It is likely that responses to high‐risk BPSD involve a combination of these immediate actions, plus those discussed above, including risk assessment and person‐centred care strategies.
Pharmacological interventions *(Red)*	The use of psychotropic medications in people with BPSD can be associated with potential harm and are not effective at controlling all symptoms; however, pharmacological interventions may need to be considered in situations of high‐risk BPSD.^2^, ^3^, ^6^, ^15^, ^16^, ^17^, ^18^ They should only be integrated into the care plan upon consultation with the family/carer(s), the person living with dementia and a GP or geriatrician, after a full discussion with all parties about the possible benefits and risks of treatment.^2^, ^3^, ^6^, ^15^, ^16^, ^17^, ^18^ Target symptoms should be identified, quantified and documented; co‐morbid conditions, such as depression, should be considered, and the dose should be initially low and titrated upwards if necessary.^3^, ^16^ If efficacy is not observed within a relatively short time frame (usually 1–2 weeks), treatment should be discontinued.^3^, ^6^, ^18^

Recommendations from these publications were extracted and arranged into a draft clinical pathway, which outlines the process for BPSD management at the different stages. The draft clinical pathway was then contextualised to the local provider in consultation with the multidisciplinary team. This process was an iterative one: discussions regarding local needs, protocols and processes were made, leading to adjustments to the clinical pathway for ease of use and clarity of management strategies and required documentation. The clinical pathway was then re‐assessed for accuracy and adherence to the evidence base, and the updated version was presented to the multidisciplinary team for further discussion. This process was repeated until agreement was reached between all parties.

## 3 RESULTS

The result is a detailed instruction guide, with two colour‐coded flow charts for quick reference, to assist staff in determining the best management and care strategies for each person living with dementia. One flow chart is specific for those with a pre‐existing antipsychotic prescription and one for those with no antipsychotic prescription. The instruction guide, also colour‐coded based on the risk levels, contains the quick reference flow charts and gives greater detail on the processes and documentation required for each step. In the instruction guide, ‘risk’ is defined as the potential for harm or danger (physical or psychosocial) to the person living with dementia, or to those around them. The degree of risk is assigned depending on three aspects that need to be assessed together: the severity of the behaviour; the context of the behaviour; and the resources available to manage the behaviour and the situations surrounding it.

The purpose of the instruction guide is to provide an evidence‐based clinical pathway with a focus on collaboration with the person, their representatives, and the broader clinical care team. The pathway can be followed by RACF staff to identify, monitor and support people living with BPSD, with specific consideration on prevention first to reduce the requirement for the use of antipsychotics or other restrictive practices within this process. The pathway incorporates local documentation and processes (these can be modified to suit each site as needed), and aims to provide a structured way to lead the care team through the development of an individual care plan. The care plan should address the person's environment and current state of physical and psychosocial health, acknowledging the individual's background (cultural, religious and social) and any known triggering actions or events as per the current Australian clinical practice guidelines.^3^ The instruction guide provides an overview of dementia, BPSD, person‐centred care, and risk assessment and levels based on the literature. The instruction guide also provides details of the pathway and the required steps included in each level of risk, including local documentation and reporting or action processes; consultation with staff, family members, the person living with dementia and any other health professionals involved in the person's care; and actioning of the personalised care plan. Processes of internal referrals (nurses, managers, allied health and chaplain) and external referrals (health‐care professionals trained in the assessment and management of BPSD, GP, pharmacist, Dementia Support Australia (DBMAS/Severe Behaviour Response Teams (SBRT)), occupational therapy, physiotherapy, environmental design services, specialist or personal medication management review, hospital outreach services, Dementia Australia and Dementia Training Australia) are included.

The instruction guide contains suggestions for different types of non‐pharmacological interventions. While the Australian clinical practice guideline^3^ did not find strong evidence supporting any particular intervention, it highlighted the need for a tailored, individualised approach to best address the range of behaviours and the many factors associated with them. More recent research provides stronger evidence for some of these approaches.^15^, ^17^, ^18^ Tips for communication with people living with dementia^19^ and the 10 Principles of Dignity in Care^20^ were added, and a BPSD consultation form was developed to step through the antecedents, behaviour and consequences of the behaviour as a tool to engage staff in an education and prevention process when a BPSD incident occurs. This includes an assessment of the need for staff education in relation to the incident.

### 3.1 The clinical pathway

In the pathway instruction manual, each stage of the pathway described below has a description of the risk and focus of the stage, the assessment(s) that should be undertaken, the development (or review) and communication of an individualised care plan, any factors or treatment recommendations, and any special considerations (i.e. current BPSD‐specific medication considerations) that are specific to that stage.

Green represents ‘prevention’; here, the person living with dementia does not have BPSD or has BPSD with effective non‐pharmacological strategies in place and is of no or low risk to themselves or others. The focus in this stage is on prevention through clinical assessment and effective non‐pharmacological strategies, or maintenance through preventative measures. On admission, or when there is any change in behaviour that indicates a change in the level of risk, a comprehensive and holistic clinical, well‐being and medical review should be conducted to detect any health problems or well‐being issues that could impact on a person's quality of life.^2^, ^6^, ^15^, ^16^ This section contains information detailing the development of a comprehensive, individual care plan (which is developed on admission as standard practice), which is communicated to all relevant stakeholders, and the importance of reviewing the care plan regularly (6‐monthly), or when changes to the person's behaviour lead to an increase in risk level.

Amber represents ‘watchful waiting’: mild‐to‐moderate BPSD is present; the person living with dementia has a mild or moderate risk of harm to themselves or to others. The focus at this stage is on the ongoing assessment of triggers identified (both known and new triggers), and implementing non‐pharmacological strategies to avoid antipsychotic use.^2^, ^3^, ^6^, ^15^, ^16^ Referrals are made to the appropriate specialist(s) as required with reviews of physical and psychosocial well‐being, and monitoring and recording of behaviours. Strategies to manage violence, aggression and extreme agitation, including de‐escalation techniques, are used.^2^, ^3^ It is essential to discuss the person's symptoms and potential triggers with staff and family to understand possible reasons for the symptoms and ways to engage the person in activities. It is also essential to have a thorough discussion with both staff and family about which strategies are working to support the person living with dementia. As the comprehensive individual care plan is developed or updated, it is communicated to all relevant stakeholders and reviewed regularly (6‐monthly or when any changes occur to the person's behaviour leading to an increase in risk level) to ensure it remains effective. A consultation guide is suggested, and links to the ‘Strategies for alternatives to restraint’ and the evidence‐based approaches for specific types of behaviour in ‘Behaviour Management: A Guide to Good Practice’^2^ are provided for further information and reading. A list of non‐pharmacological interventions supported by evidence in the Australian clinical practice guidelines,^3^ and dementia‐enabling environment resources are provided. A plan for monitoring and reviewing behaviour is developed and communicated, and a plan for escalation to specific intervention (below) is developed in case the person does not respond to the non‐pharmacological interventions, or an emergency situation occurs.

Red represents ‘specific intervention’; here, severe BPSD is present and the person living with dementia has a high risk of causing harm to themselves or to others. Pharmacological intervention is required. The specific intervention is split into two sections: (1) those not currently prescribed an antipsychotic (new prescription) and (2) those who are currently prescribed an antipsychotic (existing prescription). Pharmacological interventions should only be initiated or maintained in consultation with the family or carer(s), the person living with dementia and a GP or geriatrician, and only once certain conditions have been met.^2^, ^3^, ^6^, ^15^, ^16^, ^17^, ^18^ Conditions include, but are not limited to: consultation with and informed consent from the person, their health‐care team, carer(s) and family about the possible benefits and risks of treatment; identification, quantification and detailed documentation of target symptoms; consideration of co‐morbid conditions; and an individual antipsychotic risk–benefit analysis.^2^, ^3^, ^6^, ^15^, ^16^, ^17^, ^18^ Full documentation and tracking of target symptoms, and regular monitoring of the effectiveness of the medication on these symptoms are required, with a deprescribing plan in place in the event of a reduction in symptoms or in case of adverse effects.^3^, ^16^ Pharmacological options are based on the best available evidence and should be used within an overall comprehensive care plan that has been tailored to the person. All antipsychotic prescriptions need to be carefully monitored and reviewed every four weeks, and deprescribing should be considered at 12 weeks.^3^, ^16^, ^18^ Site‐specific emergency management is recommended in case of immediate risk.

This pathway was developed for use by nurses and care workers for the prevention or management of BPSD in RACFs with the intention of reducing antipsychotic use or any form of restrictive practices specific to BPSD. The discontinuation of antipsychotic medication should only be attempted under the guidance of a GP or geriatrician and only if the antipsychotic has been prescribed for BPSD; antipsychotic prescription for psychoses does not fall under the scope of this pathway.^18^

### 3.2 Limitations and next steps

This pathway was developed at one RACF, and while effort has been made to ensure that the pathway can be made relevant to other sites, it is currently specific to the site within which it was developed. As such, generalising it to other locations is not recommended at this stage. As the pathway was developed for the use of nurses and personal care workers, they were originally considered to be the consumer group and were involved in the development of the pathway. However, as the outcomes of the pathway will impact upon the person living with dementia and their families or carers, they will need to be involved in all future development and testing of this tool. Their lack of involvement in this stage of development is a limitation of the current pathway.

Further work is needed to refine the pathway, examine the initial staff education needs and test the feasibility and implementability of the pathway in the RACF in which it was contextualised. It will then need to be pilot‐tested for applicability and generalisability for wider dissemination throughout Australian RACFs. This clinical pathway will undergo testing in one memory support unit to assess the feasibility and implementability of the pathway. A local site champion will lead the education of staff on the use of the pathway and will facilitate and monitor its implementation with support from the research team. Medical record audits, surveys and interviews will be sought to test the data collection strategies and get feedback on the usability and acceptability of the pathway. This feedback will be used to update the documents and the assessment processes before wider pilot testing of the effectiveness of this clinical pathway commences.

## 4 CONCLUSIONS

A draft clinical pathway was developed to enhance the translation of the current best evidence into practice for the management of BPSD in RACFs. The pathway was informed by Australian clinical practice guidelines and international literature for the safe, person‐centred care of those living with dementia and experiencing BPSD in RACFs. The clinical pathway is aimed at clinical leaders and nurses in Australian RACFs with the goal of providing a structured plan of care contextualised to the local setting, which standardises care and improves the quality of life for people living with dementia. This draft clinical pathway will undergo testing for feasibility and implementability at the development site, before pilot testing for feasibility, acceptability and usefulness in multiple RACFs.

It is hoped that this pathway will assist in addressing some of the gaps highlighted in Australian residential aged care in recent years. This pathway aims to address staff knowledge and confidence in RACF processes and protocols for very early management or prevention of potential BPSD incidents, without the use of physical or medical restraint. As such, the onus is on RACF management to promote, and their staff to undertake, professional development in dementia and dementia care. This requires managerial support and staff engagement to be successful. The best ways in which to engage both groups need to be considered in any implementation process to ensure long‐term uptake of the pathway.

## CONFLICTS OF INTEREST

No conflicts of interest declared.

## Data Availability

Data sharing not applicable to this article as no datasets were generated or analysed during the current study.
